# Same‐day test and treat for infants with HIV infection: finally within reach

**DOI:** 10.1002/jia2.26016

**Published:** 2022-09-22

**Authors:** Lara Vojnov, Diane Havlir, Landon Myer, Elaine Abrams, Ilesh Jani

**Affiliations:** ^1^ World Health Organization Geneva Switzerland; ^2^ University of California San Francisco California USA; ^3^ Division of Epidemiology & Biostatistics, School of Public Health University of Cape Town Cape Town South Africa; ^4^ Columbia University New York City New York USA; ^5^ Instituto Nacional de Saúde Marracuene Mozambique

1

In 2021, there were more than 150,000 new HIV infections among children; however, only 52% of children living with HIV were on antiretroviral treatment (ART) [1]. Untreated infants and young children are at high risk for rapid disease progression and death [2, 3]; early diagnosis and rapid treatment can prevent these outcomes [3]. In 2021, however, less than 65% of HIV‐exposed infants received an infant test within the first 2 months of age [1]. The median time between sample collection to the results being received at the clinic was over 40 days in a recent systematic review of laboratory‐based, standard‐of‐care infant testing in low‐ and middle‐income countries [4]. Further, 15% of infants were known to have died during the prolonged lag time between testing and ART initiation.

Fortunately, HIV nucleic acid tests for infant diagnosis that can be performed closer to the patient and provide results on the same day of sample collection are now available and have been approved by regulatory authorities [5]. In 2016, WHO conditionally recommended the use of point‐of‐care technologies for infant diagnosis [6], a recommendation then based on low‐certainty evidence from only two diagnostic accuracy studies. Since then, there have been new data supporting the diagnostic accuracy of these tests and the clinical benefit when results are provided on the same day with linkage to immediate action—a rapid HIV test and treat approach for infants. A systematic review of 12 studies found the diagnostic accuracy of point‐of‐care technologies was greater than 98% sensitive and 99% specific [7]. In addition, a recent systematic review on the clinical impact of point‐of‐care testing identified seven studies that included 37,000 infants across 15 countries in sub‐Saharan Africa. This study demonstrated that same‐day point‐of‐care testing significantly reduced the time from sample collection to result delivery to caregivers compared to laboratory‐based testing from 35 days (95% confidence interval [CI]: 35–37) to 0 days (95% CI: 0–0) and from sample collection to ART initiation among infants testing positive from 39.5 days (95% CI: 36–44) to 0 days (95% CI: 0–1) [8]. The overall proportion of infants living with HIV initiating ART within 60 days was 90% (95% CI: 77–97) when tested at the point of care compared to 52% (95% CI: 27–76) when tested using laboratory‐based assays. Infants living with HIV tested using same‐day point‐of‐care testing were nearly nine times more likely to start ART within 60 days (odds ratio: 8.74; 95% CI: 6.6–11.6, *p*<0.001). In fact, same‐day results were returned to caregivers 97% of the time, while 51% of infants living with HIV initiated ART on the same day as sample collection versus 0% for both metrics when tested using laboratory‐based testing. Only two studies reviewed outcomes beyond treatment initiation, including retention in care and mortality, and observed minimal differences between study arms.

Same‐day point‐of‐care testing is also cost‐effective compared to standard‐of‐care laboratory‐based testing in various settings [9]. In addition, in most modelled scenarios, integrating or sharing platforms across diseases [10, 11] resulted in point‐of‐care testing being cost‐saving compared to the standard of care. In Zambia, for example, point‐of‐care testing cost US $752 less than the standard of care per additional ART initiation when sharing the devices across tuberculosis and HIV programmes.

Furthermore, an ethical and equity analysis determined that concerns about costs should not be a barrier to the adoption of point‐of‐care HIV testing for infants [12]. Given the conclusive clinical and public health evidence, scale‐up and implementation of same‐day point‐of‐care testing should be prioritized by global and national HIV programmes. This would also finally provide access parity to HIV diagnosis and ART linkage for this vulnerable population.

Based on the clear benefits of the intervention, the WHO guideline development group now strongly recommends same‐day point‐of‐care testing to diagnose HIV among infants and children younger than 18 months of age [13]. In the last 10 years, few public health interventions have shown such considerable clinical impact. Unfortunately, the COVID‐19 pandemic has resulted in setbacks to infant diagnosis and linkage to ART [14]; however, implementing and scaling up point‐of‐care testing with same‐day ART initiation could be an effective catch‐up tool. The impact of this global policy change is now dependent on programmatic action at the country level as well as on the availability of funding for implementation.

For the overwhelming clinical benefits of this intervention to be realized, clear messaging, communication and literacy considerations are urgently needed to support demand generation, scale‐up, trust and utilization, including close collaboration with community groups. Further, maximizing the impact of point‐of‐care testing will require strengthening of treatment and care services for children, including expanding efforts to identify mothers with HIV infection and infants at risk, same‐day linkage of infants to treatment and care, reliable procurement of appropriate paediatric antiretroviral formulations and high‐quality health services addressing the needs of families with HIV infection.

In many countries with a high burden of HIV infection, most infants with HIV exposure receive care at a small proportion of healthcare facilities. For example, in analysis across Malawi, Mozambique, Uganda and Zambia, 80% of infants with HIV exposure attended 32%, 33%, 12% and 10% of healthcare facilities, respectively. In other words, 10% of healthcare facilities serve 49%, 46%, 75% and 80% of HIV‐exposed infants, respectively, in these four countries. Indicating that modest procurement and focused placement of point‐of‐care technologies would affect a considerable proportion of at‐risk infants [13, 15]. Therefore, point‐of‐care technologies may not need to be procured for every healthcare facility to reach most HIV‐exposed infants.

In healthcare facilities where point‐of‐care testing cannot be implemented, alternative options must be found, including ensuring rapid and continuous laboratory‐based testing. Maintaining and improving the current laboratory‐based system will be essential to provide training and quality support to decentralized testing sites as well as to scale‐up testing for HIV viral load, tuberculosis, COVID‐19 and influenza, ideally using shared multi‐disease testing technologies.

Same‐day point‐of‐care testing has been widely recommended, implemented and expanded in other health areas, including HIV testing for adults, and when accurate, feasible and cost‐effective has led to substantial clinical and public health benefits. Achieving ambitious paediatric 95‐95‐95 targets will require novel and effective interventions: same‐day infant test and treat could finally be a reality. It is time for countries and stakeholders to act: implementing and expanding access to point‐of‐care infant testing to allow for same‐day ART is long overdue and will be a key element to achieving national and global targets.

## COMPETING INTERESTS

The authors declare no competing interests.

## AUTHORS’ CONTRIBUTIONS

LV conceived this viewpoint and wrote the first draft of the manuscript. LV, DH, LM, EA and IJ reviewed the manuscript and had full responsibility for the decision to submit it for publication.

## FUNDING

There was no funding for this work.
